# A High Soldier Proportion Encouraged the Greater Antifungal Immunity in a Subterranean Termite

**DOI:** 10.3389/fphys.2022.906235

**Published:** 2022-06-06

**Authors:** Wenhui Zeng, Danni Shen, Yong Chen, Shijun Zhang, Wenjing Wu, Zhiqiang Li

**Affiliations:** Guangdong Key Laboratory of Animal Conservation and Resource Utilization, Guangdong Public Laboratory of Wild Animal Conservation and Utilization, Institute of Zoology, Guangdong Academy of Sciences, Guangzhou, China

**Keywords:** antimicrobial peptide, phenoloxidase, entomopathogenic fungus, group immunity, innate immunity

## Abstract

Termites possess a mighty social immune system, serving as one of the key obstacles to controlling them biologically. However, the dynamic mechanism coordinating the social immunologic defense and caste distribution of the termites remains elusive. This study used the *Coptotermes formosanus* Shiraki and an entomopathogenic fungus as a host–pathogen system and experimentally manipulated a series of groups with different caste compositions of workers and soldiers. Then, the impact of demography on the behavior and innate immunity of termites was explored by analyzing the fungus susceptibility of the respective caste, efficiencies, and caste preferences of sanitary care, as well as the expression of the immune genes and phenoloxidase activity. Overall, to ensure the general health and survival of a group, the infected workers were found to sacrifice their survivorship for maintaining the soldier proportion of the group. If soldier proportion was limited within a threshold, both the survivorship of the workers and soldiers were not significantly affected by the infection. Correspondingly, the infected group with a higher proportion of soldiers stimulated the higher efficiency of a non-caste-biased sanitary care of the workers to the nestmate workers and soldiers. Moreover, the innate immunities of the infected workers were found to be more intensely upregulated in the group with higher soldier proportions. This suggested that the adjustable non-caste-biased sanitary care and innate immunity of the workers would contribute to the flexibility of the worker–soldier caste ratio in *C. formosanus*. This study, therefore, enhanced our understanding of the functional adaptation mechanism between pathogen-driven social immunity and the demography of the termites.

## Introduction

Termites are a type of social insect whose caste is precisely determined by the demographic division of labor with respect to the morphological, physiological, and behavioral characteristics of the colonies (Robson and Traniello, 2016; He et al., 2018). The subterranean termites (Rhinotermitidae) live in microbe-rich environments, and caste distribution serves as the adaptative mechanism for the survival of termites, not only improving the coordination of social immunity but also promoting individual fitness (Rosengaus and Traniello, 2001; Gao and Thompson, 2015). Disease-related selection pressures might influence the caste distribution function for enhancing the colony fitness and have remained relatively unexplored in termites (Rosengaus and Traniello, 2001). Studying the relationship between social immunity and caste distribution is highly significant for clarifying the mechanism of demographic division of labor driven by the disease in termites.

The social immune system of termites generally comprises interconnected strategies of the innate community at the individual level and behavioral defenses at the group level (Cremer, 2019). At the group level, termites demonstrate behavioral immunity mainly through the sanitary behaviors like the grooming, the burying/eating corpses of the workers, antibacterial exocrine oral secretions, carrying/attacking sick nestmates, or the external defense functions of the soldiers (Sobotník et al., 2010; Kaji et al., 2016; Cremer et al., 2018; He et al., 2018). Grooming behavior is particularly important in sanitary care as it can externally remove the pathogens from the cuticles and protects the termite from disease during the early stages of infection (Rosengaus et al., 1998; Rosengaus et al., 2000; Yanagawa and Shimizu, 2007; Chouvenc and Su, 2010; Yanagawa et al., 2011; Liu et al., 2019a). The healthy workers could identify infected termites and actively perform grooming behavior. The worker termites exposed to pathogens could mutually groom each other (Yanagawa et al., 2009, 2010, 2011; Bulmer et al., 2019). The individual immunity of the worker termites, mainly referred to as innate immunity, is endowed with a conserved insect immune system: several pathogen recognition genes like *gnbp* (Bulmer and Crozier, 2005; Bulmer et al., 2009) and a variety of antimicrobial peptides, like termicin, defensin-like peptides, spinigerin, and lysozymes have been found in the worker termites (Lamberty et al., 2001; Lee et al., 2003; Matsuura et al., 2007; Rosengaus et al., 2007; Hamilton and Bulmer, 2012; Zeng et al., 2016). Moreover, the pathogens can be embedded by the phenoloxidase cascade reaction-mediated melanization in the workers (Chouvenc et al., 2009a). However, the innate immunity of the soldiers was mainly found to be weak, and the mandibular morphology impeded the implementation of sanitary behaviors from being implemented. As a result, the soldiers failed to resist the pathogen invasion on their own (He et al., 2018). Meanwhile, the ability of the termite colony to defend itself relies not only on the number of workers. For instance, the survivorship of *Zootermopsis angusticolis* Hagen nymphs was found to be higher when exposed to the disease in small groups compared to those isolated (Rosengaus and Traniello, 2001). The soldier–worker mixed-caste groups of *Nasutitermes* spp*.* could resist the *M. anisopliae* infection better than the mono-caste groups of either caste (Rath and Andrew, 2000). The social context lies in significantly affecting the susceptibility and immune gene expressional response of the groups of termites to the pathogenic fungi (Qi et al., 2012; Gao and Thompson, 2015; Liu et al., 2019a). The immunity-associated behavior of the group as well as the individual innate immunity of termites are not demarcated but form a network structure that helps in defending against the pathogens (Stroeymeyt et al., 2018; Liu et al., 2019b). The ratio of worker to soldier in the termite colony is flexible inthe natural environment, where the soldiers constitute 5–20% of a typical insect colony (He et al., 2018). However, the behavior and innate immunity of the worker termites responsible for the formation of the new fortification that resists the pathogenic microorganism infection in the case of the changing ratio of workers to soldiers in a colony is very little known.

This study involved the wood-feeding subterranean termite *Coptotermes formosanus* Shiraki, which is a globally distributed economic pest (Blumenfeld et al., 2021). These termites are endowed with a unique and social immune defense system that strongly limits their biological control (Bulmer et al., 2009; Chouvenc and Su, 2010). Studies on the immune mechanism of *C. formosanus* have been so far focused mainly on the effect of population size as well as the grooming behavior of the workers on the survival rate, identification of pathogenic fungi of termites (Yanagawa and Shimizu, 2007; Yanagawa et al., 2011; Yanagawa et al., 2008; Yanagawa et al., 2009; Yanagawa et al., 2010; Husseneder, 2010), and the expression of the defensive genes in response to the pathogen infection (Hussain et al., 2013; Mitaka et al., 2017a). The entomopathogenic fungus *Metarhizium anisopliae* Sorokin (MA) is a widely used agent for biologically controlling pests and is ideally used for experimentally studying the immunity of social insects (Liu et al., 2015). Here, the *C. formosanus* and the *M. anisopliae* were used as a host–pathogen system and a series of groups with different caste compositions of workers and soldiers were experimentally manipulated. First, the survivorships of the respective caste in each worker–solider composition were analyzed. Based on the results of the survival assay, the increase in the soldier proportion was hypothesized for encouraging the efficiency of sanitary care and the innate immune level of the workers. This suggested that the group survival of termites is not significantly affected by a certain range of variation in the caste ratio. Therefore, the number of epidermal conidia was observed and counted for assessing the efficiencies and caste preferences of the sanitary behavior of workers. Then, the innate immune levels (immune enzyme activity and immune gene expression of workers) were analyzed for exploring the impact of the caste ratio (worker/soldier) in coordinating the behavior and innate immunity of the termites. This study enhanced our understanding of the functional adaptation mechanism between the social immunity and demography distribution of termites and enriched us with the theoretical data for developing new control methods in the future based on the immune mechanisms of termites.

## Materials and Methods

### Termite Collection and Maintenance

This study collected three colonies of the subterranean termite *C. formosanus* from the Dafu Mountain Forest Park of Guangzhou, China. These colonies were maintained in three plastic boxes (30 cm × 25 cm × 20 cm) and fed wet pinewood, respectively. The plastic boxes were placed in the artificial climate incubator (Jiangyunguangdian PRX-900, Beijing, China), rearing the termites in complete darkness, and at a temperature of 27 ± 1°C, and 70 ± 5% relative humidity (RH).

### Preparation of the Fungal Conidial Suspensions

This study selected one entomopathogenic fungus, *M. anisopliae* (strain GDM 3.528, from Guangdong Microbial Culture Collection Center, China) which was cultivated on potato dextrose agar (PDA, HuanKai Microbial, Guangzhou, China) at 25 ± 1°C for approximately 10 days. Then, the conidia were harvested by washing each plate with a sterile 0.1% Tween-80 solution (TW 80) and stored at 4°C for no more than 10 days before using for infectivity trials. The number of conidia was then counted using a Thoma hemocytometer (SAIL BRAND, Yancheng, China) and diluted to the required concentration using 0.1% Tween-80.

### Survival Assay

The survival assay established the range of caste ratios across the groups of infected and control termites. The caste composition of the worker/soldier was experimentally manipulated using a fixed group size (*n* = 20) to: 18:2, 16:4, 14:6, 12:8, 10:10, and 0:20. In relevant studies (unpublished data), a small population of *C. formosanus* (*n* ≤ 30), infected with MA conidia at a concentration of approximately 3 × 10^7^ conidia/ml was found to well distribute the mortality curve of the *C. formosanus* group within a week (avoid termite death too fast or pathogenic efficiency too low). The workers and soldiers from each group were inoculated with the conidia by submerging them into the conidial suspension (3 × 10^7^ conidia/ml) of *M. anisopliae* separately within a microcentrifuge tube, with gentle swirling for 5 s, and then dried on a filter paper (Hussain et al., 2013). The control groups were established using sterile 0.1% TW 80 following a similar procedure. The infected and control groups were reared separately in 3.5 cm diameter Petri dishes using a moist filter paper for 7 days (maintained at 27 ± 1°C, 70 ± 5% RH in darkness). The survivorship of each group was monitored daily for 7 days and the dead termites were removed after data recording. For the survival assay, nine biological replicates were considered per group. The number of noninfectious lethal individuals in the infected groups was corrected by subtracting the number of dead termites in the control group.

### Expression of Immune Genes

The infection method was similar to that described in the survival analysis. The transcription amount of the antimicrobial peptides termicin (*tem*) and lysozyme (*lys*) were quantified. These peptides have been previously implicated in defending termites and insects in general against the pathogenic fungi through conserved immune pathways (Hamilton and Bulmer, 2012; Hussain et al., 2013). The survival analysis indicated the survivorships of the workers among the groups 18:2, 16:4, and 14:6 to be not significantly different. The innate immune response of the termites caused by fungal penetration through the epidermis into the hemocoel mainly occurs between 36 and 96 h postinfection (Chouvenc et al., 2009a). Thus, the workers from these three groups were selected for assessing the degree of innate immune response on the 3rd and 4th day postinfection. The total RNA was extracted from 10 workers collected from the three replicates of each infected and control group using a Total RNA Kit II R6934 (Omega, Guangzhou, China). Equal quantities of RNA (1.0 µg) were used as a template for generating cDNA using the EasyScript^®^ One-Step gDNA Removal and cDNA Synthesis SuperMix (TransGen, Beijing, China). The quantitative real-time PCR (qPCR) was performed using the TransStart^®^ Green qPCR SuperMix (TransGen, Beijing, China) according to the manufacturer’s instructions. The control group was used as a calibrator for calculating the amount of relative expression. The relative expression levels for the specific genes, relative to the reference gene *GADPH* (Gao et al., 2012), were calculated using the 2^−ΔΔCT^ method (Livak and Schmittgen, 2001). Three biological replicates were considered for each group and all the primers are detailed in Table 1.

**TABLE 1:** The primers were used for quantitative real-time PCR. *tem*, *lys,* and *GADPH* symbolize the genes of termicin, lysozyme, and glyceraldehyde-3-phosphate dehydrogenase (reference gene), respectively.

Gene name	Primers (5′-3′)	GenBank accession no.
*tem*	AGG​CTA​GTC​ATT​TGG​CAG​GAA​GTT	JX311465.1
	CAG​TGG​TGG​TGG​GGA​TGA​TAG​AAA	
*lys*	CGC​GTG​GCT​ATT​AAA​GAA​CTG​ATT	JX876647.1
	GAG​GGT​AAG​GGG​TAT​TGG​TTT​CAC	
*GADPH*	TTG​GGT​TGG​GAT​GGT​TTA​GG	KC740815.1
	AGC​CAC​ATC​ACC​AAT​ACG​ATT​A	

### The Phenoloxidase Activity Assay

Corresponding to the gene expression assay, the workers were sampled on 3 and 4 d after treatment with *M. anisopliae* and TW 80, using the same grouping and infection method. The Phenoloxidase activity was determined using the Tyrosinase Activity Detection Kit (Solarbio, Beijing, China) according to the manual instructions with minor modifications. Ten termites were collected from three replicates of each infected and control group, immobilized on ice, and dissected into two parts: salivary gland/foregut (FS) + midgut(M) and epicuticle (C, including fat body and hemolymph). The dissected tissues were treated with the extraction buffer of the kit, homogenized by a frozen steel ball homogenizer (Scientz-48L, Suzhou, China), and then centrifuged at 4°C for 10 min. The supernatants were used as the crude extracts. The reaction mixtures comprised 20 µl of crude extract and 100 µl of Kit L-DOPA solution and were incubated at 30°C. The value of light absorption was measured every 30 min and lasted for 2 h at 490 nm on a microplate reader (Perkin Elmer, Waltham, MA, United States). To calculate the enzyme activity, the value of the largest change of light absorption was assessed at a 30 min reaction interval. One unit (U/mg protein) of phenoloxidase activity was defined as the amount of enzyme capable of catalyzing the formation of 1 nmol DOPA pigment per mg tissue protein per minute. The concentrations of protein were determined using the Bradford Protein Assay kit (Beyotime, Shanghai, China). There were three biological replicates for each group.

### Detection of the Conidia on the Termite Epicuticle

Sanitary care is an effective behavioral adaptation of the workers for removing conidia from the epicuticle of the nestmate termites. The efficiency and caste preference of sanitary behavior were assessed in the manipulated small groups comprising only infected termites (worker and soldier) or a mixture of uninoculated (worker) and infected termites (worker and soldier). The uninfected workers were considered for analyzing whether the uninfected worker possessed a hygienic preference between the infected soldiers and workers. The caste compositions of the infected worker/infected soldier (A): 2:0, 2:1, 2:2, 2:4, 0:2; infected worker/uninfected worker/infected soldier/: 1:1:2, 1:1:4 (B) were experimentally manipulated. The number on the scale represented the number of termites in the experiment. To meet the requirements of microscopic counting, the spores should have good dispersion and should not overlap after adhesion to the termite epidermis. The conidial suspension was diluted to a concentration of 2 × 10^5^ conidia/ml. The conidia on the epicuticles were detected by surface-labeling the conidia of *M. anisopliae* by adding 100 µl 18909 Calcoflour White Stain (Calcofluor White M2R 1 g/L and Evans blue 0.5 g/L, Sigma-Aldrich, United States) per milliliter of the conidial suspension. Before infection, the termites were placed on ice for 3 min ensuring that the termites are in a state of coma so that they do not swallow the fluorescent conidial fluid. Then, the termites were infected with the Calcoflour White-labeled conidial suspensions as described in the survival assay, and chilled at 4°C for 20 min to prevent immediate grooming, and washed once in TW 80 to remove the nonattached conidia. The uninfected workers were labeled a small dot at the end of the dorsal epicuticle with a red marker pen and chilled at 4°C for 20 min. The infected and the uninfected termites were grouped and reared separately in a 24-well microtiter plate containing a filter paper disc soaked in distilled water at 27 ± 1°C, 70 ± 5% RH in darkness. The preliminary experiment results showed that under the aforementioned experimental conditions, the experimental worker and soldier termites took no more than 8 h to completely clean the conidia on their dorsal epicuticle. Thus, the termites were sampled from each group 0 and 4 h postinfection and stored at −20 °C for later observations. The termites were examined under a fluorescent microscope (EVOS FL Auto, Life Technologies, United States) at ×200. A total of five defined dorsal epicuticle sites of each termite (head, thorax, 2nd, 4th, and 6th of abdominal segments) surface were examined for attaching the conidia (Yanagawa and Shimizu, 2007). There were 18 replicates for each group.

### Statistical Analyses

All the data were statistically analyzed using the SPSS 19.0 (SPSS Inc., Chicago, IL, United States) software. The survival assay was calculated with a log-rank test (Mantel—Cox) for multiple testing (*p* < 0.05). The events with a survival time of less than or equal to 7 days were defined as 1, while events with a survival time of more than 7 days belonged to right censoring data and were defined as 0. The differences in the mortality rate between the two groups were analyzed using the independent Mann–Whitney *u*-test (*p* < 0.05). The data of the total number of epicuticle conidia, immune gene expression, and phenoloxidase activity among the groups were analyzed using the one-way ANOVAs, LSD (*p* < 0.05). The data were represented as the mean and standard error of the mean (SEM).

## Results

### Survival Assay

**FIGURE 1:**
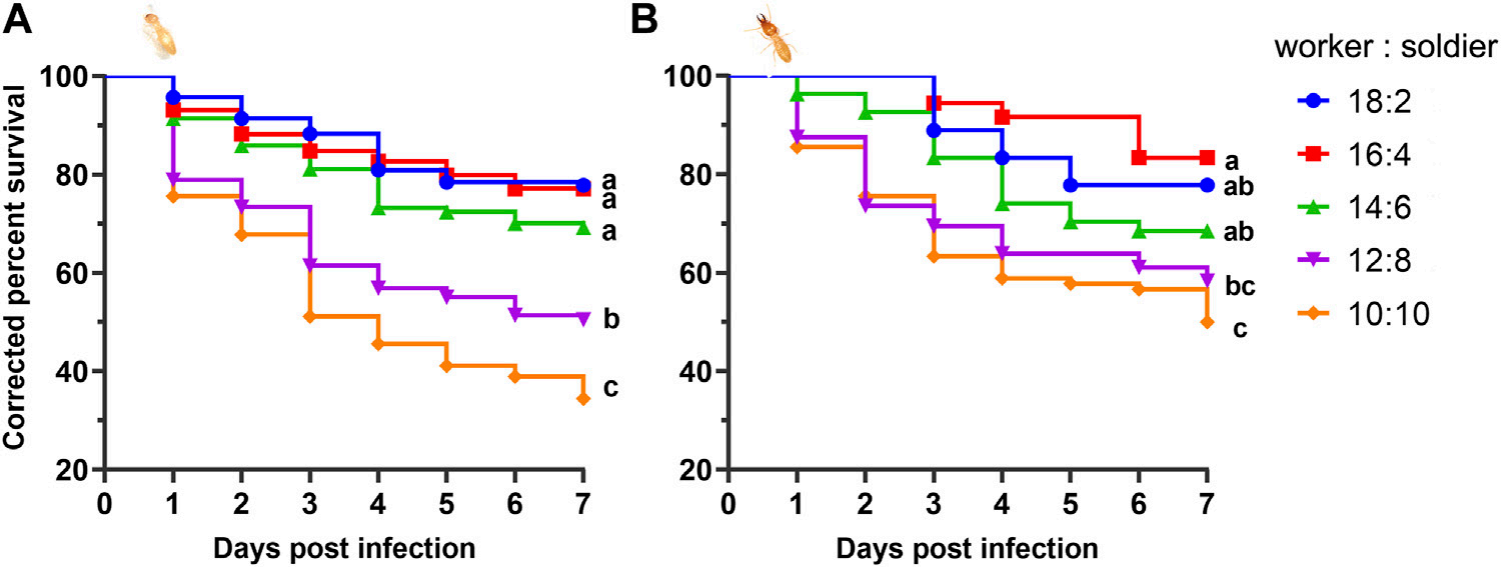
survival curve of the *Metarhizium anisopliae-*infected worker **(A)** and solider **(B)** *Coptotermes formosanus* in different compositions of worker and soldier ratio under the fixed group size (*n* = 20). The numbers in the legend indicate the specific number of workers and soldiers. The *p*-values were calculated using the Log-rank (Mantel—Cox) test (*p* < 0.05). Nine biological replicates were performed for each composition.

**FIGURE 2:**
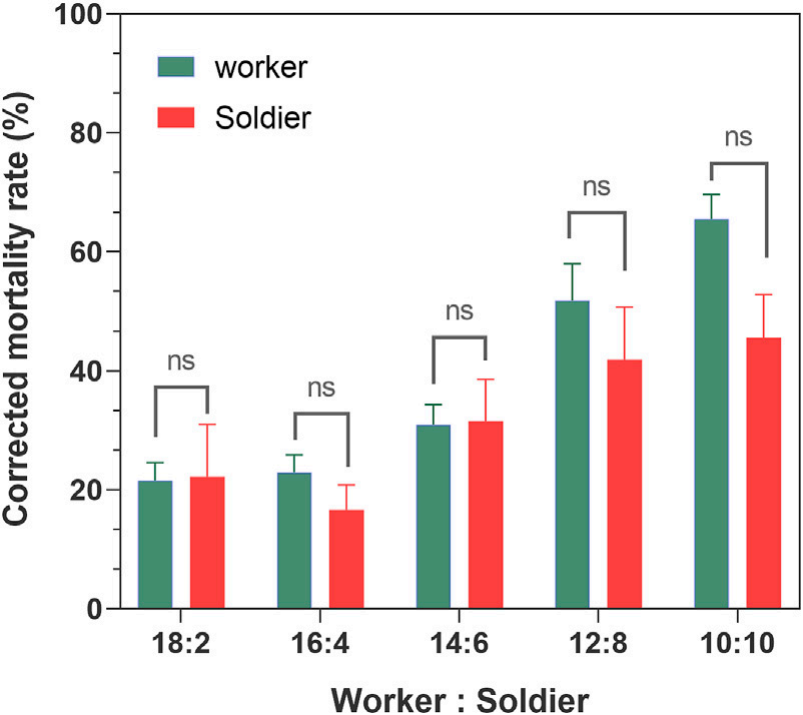
Mortality rate of the *Metarhizium anisopliae* −7 d postinfected worker and soldier *Coptotermes formosanus* in different compositions of worker and soldier ratio under fixed group size (*n* = 20). The numbers in the abscissa represent the numbers of infected workers and infected soldiers in each group, respectively. Differences between the mortality of the worker and soldier within group were statistically analyzed using the independent Mann–Whitney *u*-test (*p* < 0.05). Data represent the mean ± standard error of the mean (SEM).

**FIGURE 3:**
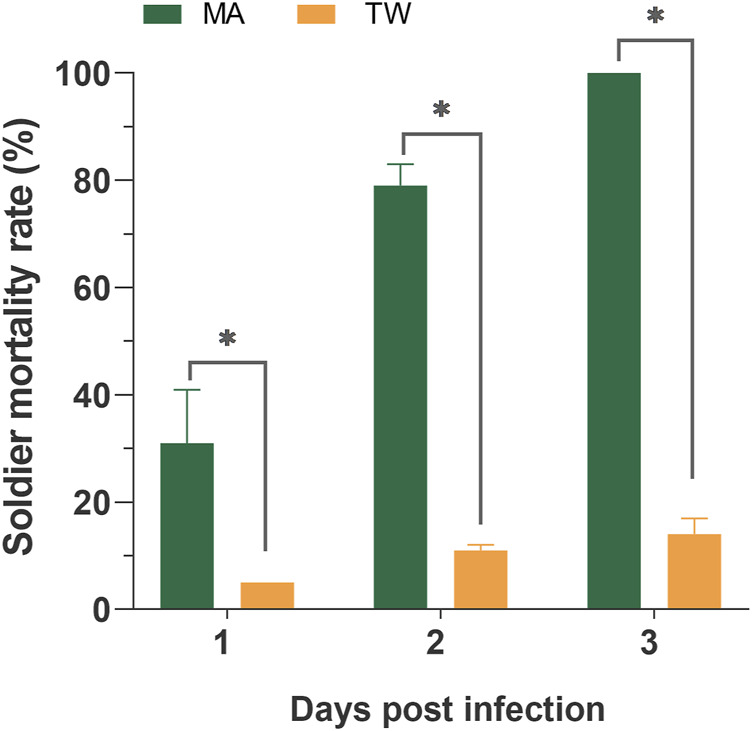
Mortality of the *Metarhizium anisopliae-*infected soldier *Coptotermes formosanus* in the mono-caste group (group size = 20). Data represent the mean ± standard error of the mean (SEM). Differences between the mortality of the infected and control groups were statistically analyzed using the independent Mann–Whitney *u*-test (*p* < 0.05). MA, *M. anisopliae*-infected group; TW, Tween-80-treated control group.

Within 7 days, the average survival rate of each mixed-caste composition treated with TW 80 (control) was more than 95%, which met the control group. The survivorship of each control treatment was not significantly affected by the caste ratio set in the experiment (Supplementary Figure S1). The number of deaths in each control group was used for correcting the number of noninfectious deaths. In the case of the fixed group size (size = 20), the survival curves of the infected workers in each group were close to each other (*p* > 0.05) when the caste ratios (worker/soldier) did not exceed 14:6. Once the number of soldiers reached eight, the survivorship of workers started being to be significantly affected and showed a sharp decrease (*p* < 0.05). There were no significant differences in the survivorship of the soldiers among the groups 18:2, 14:16, and 12:8 (*p* > 0.05), and between the group 12:8 and 10:10 (χ^2^ = 0.606, *p* = 0.436) (Figure 1). Moreover, although the mortality within group between soldiers and workers in each composition was found to be not significantly different (*p* > 0.05), the mortality of workers showed a trend of higher than that of a soldier in the group 12:8 and 10:10 (Figure 2). The mono-caste group comprising only soldiers (size = 20) completely died within 3 d post-MA infection (Figure 3).

### Expression of Immune Genes

**FIGURE 4:**
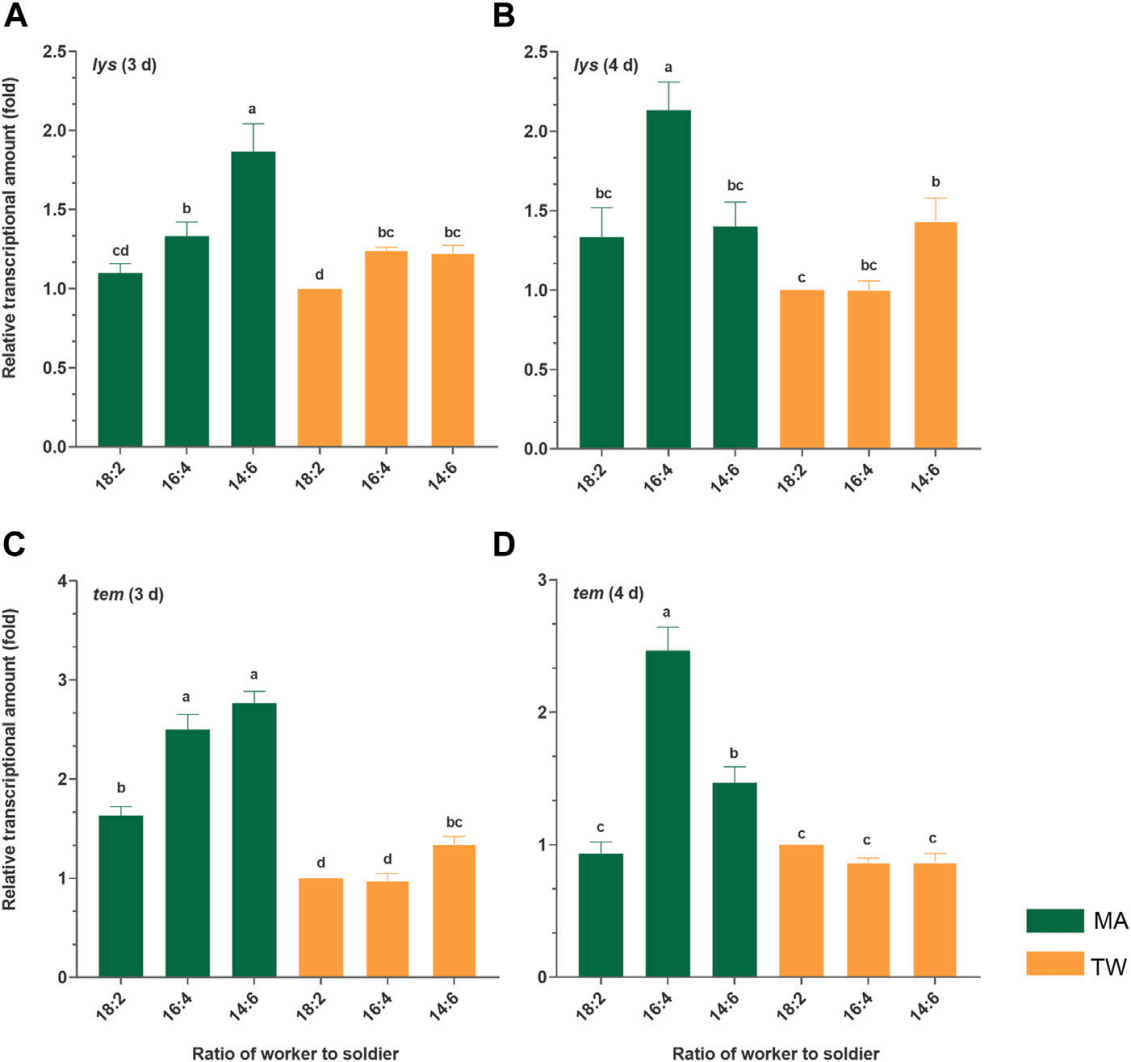
Relative transcriptional amount of the two immune genes in the *Metarhizium anisopliae-*infected worker *Coptotermes fomosanus*. The termites of the group 18:2 treated with 0.1% Tween-80 solution were used as controls, and the relative expression levels of the specific genes were normalized to that of the reference gene, *GADPH*. The error bars represent the mean ± SEM. Different letters indicate significant differences among the six sets of data within a time phase (one-way ANOVAs, LSD, *p* < 0.05). The letters “**(A–D)**” at the top of the picture represent the identifiers of the four histograms, respectively. *tem*, termicin; *lys*, lysozyme; MA, *M. anisopliae*-infected group; TW, Tween-80-treated control group.

Both the antimicrobial peptide genes were significantly (*p* < 0.05) upregulated in different degrees 3 d and/or 4 d postinfection. Except for the basal expression of the *tem* gene 4 d posttreatment, both at the basal and immune induction conditions, the expression level of *lys* and *tem* in the group with the lowest soldier proportion (18:2) were significantly (*p* < 0.05) lower than those in the group 16:4 or/and 14:6. Furthermore, 3 d postinfection, the expression of *lys* showed a significant increasing trend (*p* < 0.05) with the increase in the number of soldiers (18:2 < 16:4 < 14:6, *p* < 0.05). The expression of *tem* 3 d postinfection also showed an increasing trend with the increase in the number of soldiers: there were significant differences between 18:2 and 16:4 groups (*p* = 0.000), and between 18:2 and 14:6 groups (*p* = 0.002) were significant. In general, the increase in the proportion of soldiers was found to significantly promote the expression of the immune effectors (*tem*, *lys*) of the workers at the basal and immune induction conditions (Figure 4).

### The Phenoloxidase Activity

The activity of phenoloxidase is a reflection of the actual intensity of the individual innate immunity to the termites, therefore, it was assessed. To correspond to the upregulation phase of immune effector genes *tem* and *lys*, the tissue phenoloxidase activities of the workers were measured 3 d and 4 d postinfection.

**FIGURE 5:**
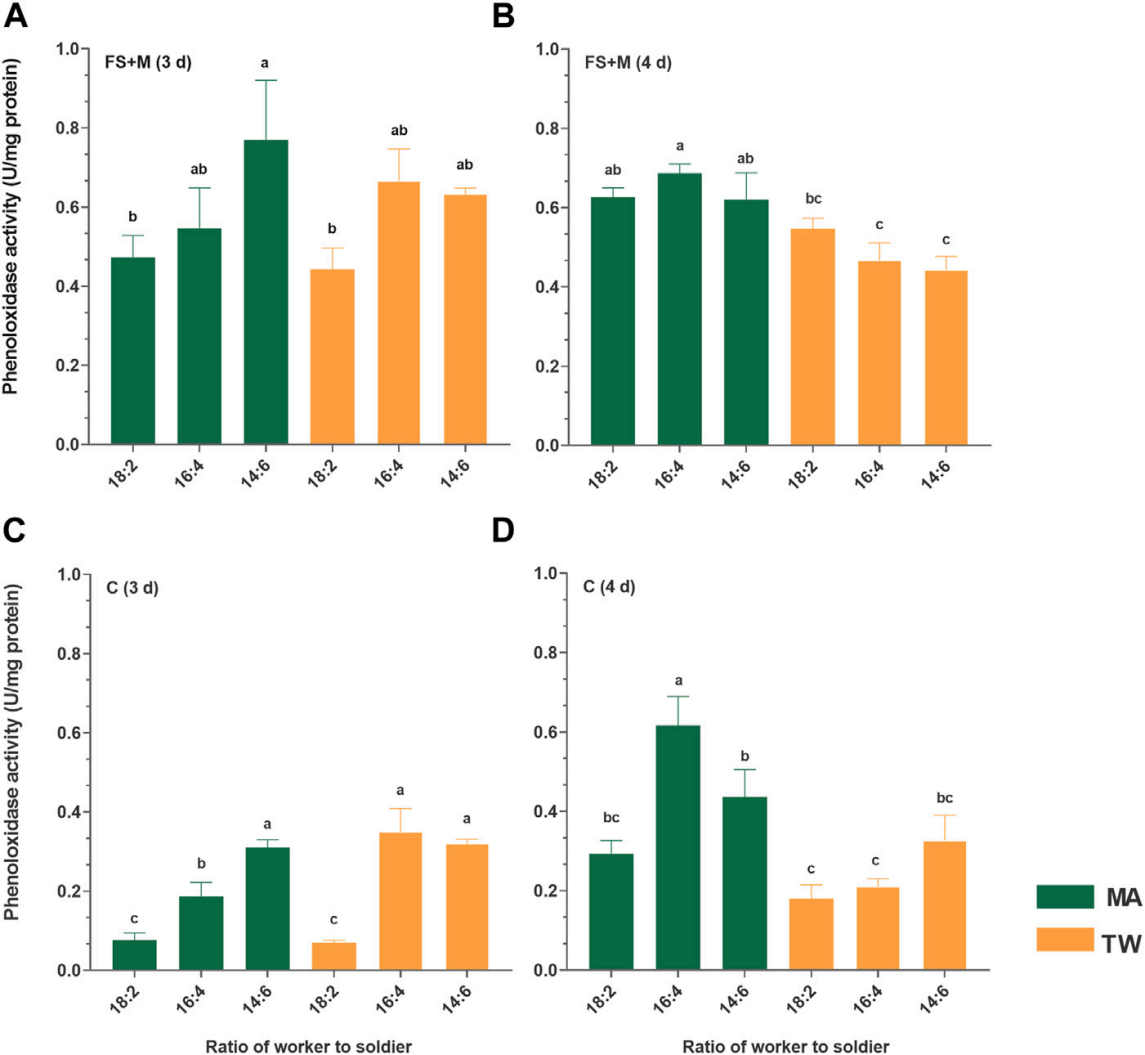
Phenoloxidase in the foregut/salivary gland + midgut (FS + M) and cuticle **(C)** of the worker *Coptotermes fomosanus*. Error bars represent mean ± SEM. Different letters indicate significant differences among the six sets of data (one-way ANOVAs, LSD, *p* < 0.05). The numbers before and after the colon in the abscissa represent the numbers of workers and soldiers in each group, respectively. The letters “**(A–D)**” at the top of the picture represent the identifiers of the four histograms respectively. MA, *Metarhizium anisopliae*-infected group; TW, 0.1% Tween-80-treated control group; FS + M, foregut/salivary gland + midgut; C cuticle (contained hemolymph and fat body).

For the FS + M and C, MA was found to be generally upregulated of the phenoloxidase activity 4 d postinfection: there were significant upregulations (comparing to TW 80 control) in the groups 16:4 (*p* = 0.002) and 14:6 (*p* = 0.01) of FS, and in group 16:4 (*p* = 0.000). Three days postinfection, the activity of the phenoloxidase in both the FS + M and C were found to show an increasing trend with the increase in the soldier proportion: the difference between the infected group 18:2 and 14:6 in the FS + M (*p* = 0.03), and among the groups 18:2, 16:4, and 14:6 in the C (*p* < 0.05) were significant. Moreover, the basal phenoloxidase activities (TW 80 control) of the cuticle in the 16:4 (*p* = 0.02) and 14:6 (*p* = 0.000) groups were also found to be significantly higher than that in the group 18:2 3 d posttreatment. Both 3 d and 4 d posttreatment, the difference in the basal phenoloxidase activities of the FS + M among the groups 18:2, 16:4, and 14:6 were not significant (*p* > 0.05) (Figure 5).

### Efficiency and Caste Preference of Sanitary Care

**FIGURE 6:**
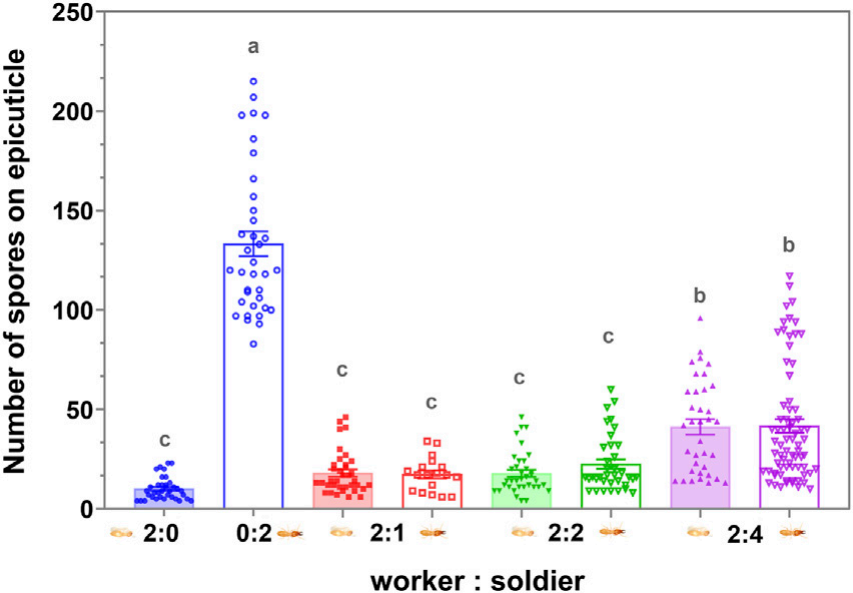
Attachment and persistence of the *Metarhizium anisopliae* conidia on the termite cuticle 4 h postinfection. Data represent the mean ± standard error of the mean (SEM). A total of five defined dorsal epicuticle sites of each termite (head, thorax, 2nd, 4th, and 6th of abdominal segments) surface were counted for the attachment of the conidia. The numbers in the abscissa represent the numbers of infected workers and infected soldiers in each group, respectively. The data columns with different colors symbolized the different soldier and worker compositions. Groups consisting only of workers or soldiers were symbolized by the blue. The data column of the solid color block indicated workers, and the hollow data column indicated the soldier. Different letters indicate significant differences among the eight sets of data (one-way ANOVAs, LSD, *p* < 0.05).

**FIGURE 7:**
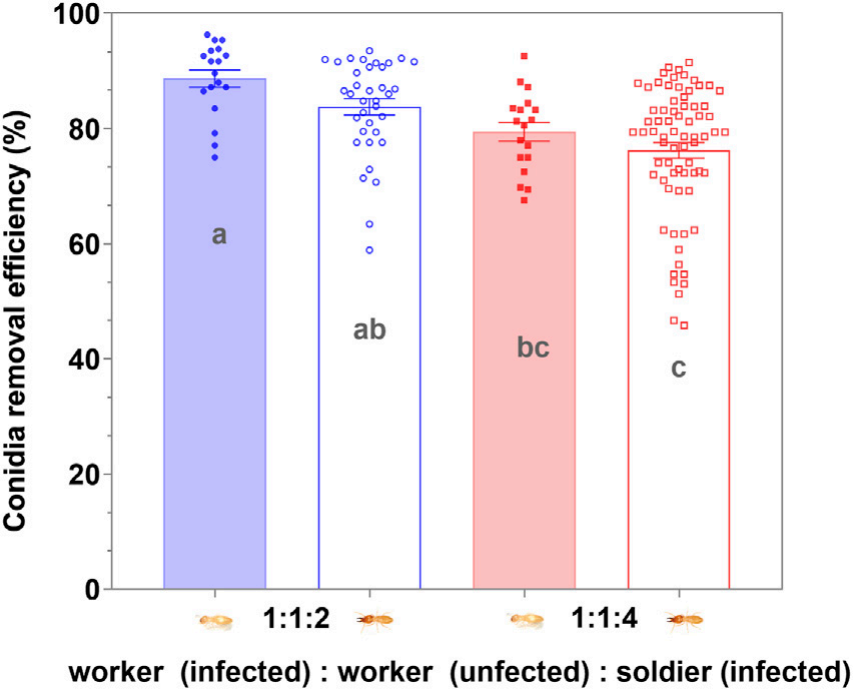
Removal efficiency of the *Metarhizium anisopliae* conidia on the cuticle by workers post 4 h infection. Data represent the mean ± standard error of the mean (SEM). The numbers in the abscissa represent the numbers of infected workers, uninfected workers, and infected soldiers in each group, respectively. A total of five defined dorsal epicuticle sites of each infected termite (head, thorax, 2nd, 4th, and 6th of abdominal segments) surface were counted for the attachment of conidia. The data columns with different colors symbolized the different soldier and worker compositions. Groups consisting only of workers or soldiers were symbolized by the blue. The data column of the solid color block indicated workers, and the hollow data column indicated the soldier. Different letters indicate the significant differences among the four sets of data (one-way ANOVAs, LSD, *p* < 0.05).

There was no significant difference in the number of conidia on the exocuticle between the workers and soldiers in the respective groups (*p* > 0.05), 4 h after exposure to the MA conidia. The increase in the proportion of the soldier had no significant effect on the degree of clearing of the conidia on the cuticle of the workers and soldiers (*p* > 0.05) when the proportion of the soldier was no more than 50% (Figure 6). When the uninfected workers were involved in the group, the efficiency of clearing the cuticular spores on the infected workers and soldiers was not significantly different (*p* > 0.05) (Figure 7). The increase in the proportion of soldiers promoted the sanitary speed of the workers, but if the proportion of soldiers exceeded the upper limit of the sanitary speed of the workers, the degree of the clearing was significantly decreased within the observed time (Figures 6, 7).

## Discussion

Termites are social insects living with numerous microorganisms in the surrounding. To counteract these potential pathogens, the termites have evolved a social immune defense line comprising individual and colonial immune systems (Cremer, 2019; Liu et al., 2019b). The demographic distributions of the social insects are found to affect the ability of the groups to organize an integral immune defense (Gao et al., 2011; Cremer et al., 2018). This study explored the adaptive response of the group and innate immunity of the *C. formosanus* workers to the variation in the worker–soldier ratio for the first time.

The termite soldiers are not efficient groomers due to the lack of specialized mandibles, so the survival of the soldiers was found to greatly depend on the sanitary behavior of the workers (Rosengaus et al., 2000; Qi et al., 2012). Here, the attachment of the conidia to the cuticle surface of *C. formosanus* soldier was found to depend on the workers removing it. Furthermore, there was no significant difference in the efficiency of sanitary care among the nestmates workers compared to the efficiency of the workers performed on the soldiers, irrespective of the involvement of the uninfected workers (Figures 6, 7). These results implied that the uninfected/infected workers of *C. formosanus* implemented sanitary care to the infected soldiers or their infected nestmate workers with no caste priority. The termites might discriminate the fungal contact nestmates by the fungal characteristics, and then provide sanitary care to the infected individuals (Yanagawa et al., 2011; Yanagawa et al., 2009; Yanagawa et al., 2010; Bulmer et al., 2019); or the infected termites might actively secrete the signal substances, to attract nestmates for cleaning them (Davis et al., 2018). The head glands of the termite soldiers were found to secrete hormones for arresting the workers for feeding or other auxiliary functions (Mitaka et al., 2017b). Based on the existing studies, the major mechanism of this no caste-bias sanitary care remains unexplained. But it was found that although the mono-caste group of soldiers was highly susceptible to *M. anisopliae* (Figure 3), the survivorships of the soldiers were found to be a not significant difference (*p* > 0.05) with workers within groups, and even showed higher (*p* > 0.05) than the survivorship of the worker in group 12:8 and 10:10 (Figures 1, 2). The workers significantly sacrificed their survival when the proportion of soldiers exceeded a threshold for maintaining the survival rate of the soldiers (Figures 1, 3). Social immunity signifies the production of colony-level disease avoidance, resistance, and tolerance (Cremer et al., 2018). Thus, these results suggested that the non-caste priority characteristics of the sanitary care and self-sacrifice performed by the workers are important for protecting the work–soldier ratio in a colony from huge fluctuations caused by pathogens.

Moreover, the social immune system of the social insects comprises both the group behavior immunity and individual innate immunity,and the regulation of these two defense systems is correlated with each other (Cremer, 2019). Termites cannot completely prevent the pathogen infection depending on social behavior alone, especially when the pathogen titer is high (Rosengaus et al., 1998). Once the pathogens have successfully invaded the body cavity of the termite, the subsequent internal infection tends to switch on the individual innate immunity, inducing the upregulation of the immune genes and antifungal activities (Thompson et al., 2003; Rosengaus et al., 2007; Liu et al., 2015; Liu et al., 2019a; Zeng et al., 2016). The survivorships of the workers among the groups 18:2, 16:4, and 14:6 were found to be not significantly different (Figure 1). Corresponding to these groups, the group with a higher proportion of soldiers had a significantly greater degree of upregulation of the immune effectors (lysozyme and termicin) of the workers after pathogenic fungal infection (Figure 4). Lysozyme and termicin belong to the antimicrobial peptides, which help the termites in resisting the pathogenic microorganisms (Fujita et al., 2002; Matsuura et al., 2007; Hamilton and Bulmer, 2012). In addition, three days postinfection, the phenoloxidase activity (including fat body and hemolymph) of *C. formosanus* showed a significant increase in proportion to the soldier number (Figure 5). The termites can convert the phenolic compounds into quinones by activating the phenoloxidase and inducing the melanization of the pathogen surfaces (Chouvenc et al., 2009b). Combining the results of the survival analysis, it is obvious that the fungus-infected *C. formosanus* stabilize the group survival in different worker–soldier caste ratio by simultaneously regulating the efficiency of sanitary care and the innate immune level of the workers.

Furthermore, previous studies have indicated that the soldiers and nymphs in combination are important for defending the colony against the pathogens (Rosengaus and Traniello, 2001; Fefferman et al., 2007; Qi et al., 2012). The termite soldiers externally defend the colony by removing or guarding the cadavers away from the population and secreting the defensive chemicals (Tian and Zhou, 2014; Kaji et al., 2016). Generally, the natural ratio of the workers to soldiers in the termite population ranges between 5 and 20% (He et al., 2018). Since the social immune response of termites is limited by the caste function, the natural worker–soldier ratio is likely the result of optimizing the populational immunologic defense ability (Qi et al., 2012; Cremer, 2019). The basal expression (noninfected control) of the antimicrobial peptide genes (Figure 5) and activities of the epidermic phenoloxidase (Figure 6) in the termites were found to be also significantly enriched by an increase in the proportion of the soldiers. The phenoloxidase activity was significantly affected by the nesting and foraging ecologies, constituting a type of immune adaptation to ecology and group living (Rosengaus et al., 2010; Rosengaus and Reichheld, 2016). Thus, the sacrifice of the workers and the non-caste preferential sanitary care of the *C. formosanus* infected/uninfected workers towards the nestmate-infected workers and soldiers was also a reflection of a type of immune adaptation to socialized survival habit. Alternately, although the adjustment efficiency of sanitary care and innate immune levels of the workers are limited, they impart certain flexibility to the caste distribution of *C. formosanus* conferring the termites with the ability to adapt to the changes in their living environment, which suggested an immune strategy to maintain the survival of termites at colony-level.

In conclusion, this study investigated the influence of the worker–soldier caste ratio of the termites on the group susceptibility to the pathogenic fungi, efficiency and caste preference of the behavioral immune, and innate immune response. The results from the present study demonstrated that the efficiency of sanitary care of the workers toward the infected nestmate workers and soldiers was not caste-biased. Moreover, the pathogenic fungi could induce the higher efficient sanitary care and innate immunity of the workers in the group with a higher soldier proportion. This adjustable social immunity contributes to the flexibility of the worker–soldier caste distribution in *C. formosanus* within a certain range*.* Based on the colony-level disease resistance was found to also reflect the optimization of the caste function differentiation and demographic distribution of the social insects under the pathogen-related derived evolutionary pressure. This study, therefore, provides novel data for uncovering the co-evolutionary mechanism of distributing the social insect demographic functions and determining their immune defense adaptability.

## Data Availability

The original contributions presented in the study are included in the article/Supplementary Material; further inquiries can be directed to the corresponding author.

## References

[B1] Blumenfeld A. J. Eyer P.-A. Husseneder C. Mo J. Johnson L. N. L. Wang C. (2021). Bridgehead Effect and Multiple Introductions Shape the Global Invasion History of a Termite. Commun. Biol. 4 (1), 196. 10.1038/s42003-021-01725-x 33580197 PMC7881189

[B2] Bulmer M. S. Bachelet I. Raman R. Rosengaus R. B. Sasisekharan R. (2009). Targeting an Antimicrobial Effector Function in Insect Immunity as a Pest Control Strategy. Proc. Natl. Acad. Sci. U.S.A. 106 (31), 12652–12657. 10.1073/pnas.0904063106 19506247 PMC2722268

[B3] Bulmer M. S. Crozier R. H. (2005). Variation in Positive Selection in Termite GNBPs and Relish. Mol. Biol. 23 (2), 317–326. 10.1093/molbev/msj037 16221893

[B4] Bulmer M. S. Franco B. A. Fields E. G. (2019). Subterranean Termite Social Alarm and Hygienic Responses to Fungal Pathogens. Insects 10 (8), 240. 10.3390/insects10080240 PMC6723859 31387197

[B5] Chouvenc T. Su N.-Y. (2010). Apparent Synergy Among Defense Mechanisms in Subterranean Termites (Rhinotermitidae) against Epizootic Events: Limits and Potential for Biological Control. Econ. Entomol. 103 (4), 1327–1337. 10.1603/ec09407 20857744

[B6] Chouvenc T. Su N.-Y. Robert A. (2009a). Cellular Encapsulation in the Eastern Subterranean Termite, *Reticulitermes Flavipes* (Isoptera), against Infection by the Entomopathogenic Fungus Metarhizium Anisopliae. J. Invertebr. Pathology 101 (3), 234–241. 10.1016/j.jip.2009.05.008 19463828

[B7] Chouvenc T. Su N.-Y. Robert A. (2009b). Inhibition of Metarhizium Anisopliae in the Alimentary Tract of the Eastern Subterranean Termite *Reticulitermes Flavipes* . J. Invertebr. Pathology 101 (2), 130–136. 10.1016/j.jip.2009.04.005 19426734

[B8] Cremer S. Pull C. D. Fürst M. A. (2018). Social Immunity: Emergence and Evolution of Colony-Level Disease Protection. Annu. Rev. Entomol. 63, 105–123. 10.1146/annurev-ento-020117-043110 28945976

[B9] Cremer S. (2019). Social Immunity in Insects. Curr. Biol. 29 (11), R458–R463. 10.1016/j.cub.2019.03.035 31163158

[B10] Davis H. E. Meconcelli S. Radek R. Mcmahon D. P. (2018). Termites Shape Their Collective Behavioural Response Based on Stage of Infection. Sci. Rep. 8, 14433. 10.1038/s41598-018-32721-7 30258216 PMC6158180

[B11] Fefferman N. H. Traniello J. F. A. Rosengaus R. B. Calleri D. V. (2007). Disease Prevention and Resistance in Social Insects: Modeling the Survival Consequences of Immunity, Hygienic Behavior, and Colony Organization. Behav. Ecol. Sociobiol. 61 (4), 565–577. 10.1007/s00265-006-0285-y

[B12] Fujita A. Minamoto T. Shimizu I. Abe T. (2002). Molecular Cloning of Lysozyme-Encoding cDNAs Expressed in the Salivary Gland of a Wood-Feeding Termite, *Reticulitermes Speratus* . Insect Biochem. Mol. Biol. 32 (12), 1615–1624. 10.1016/s0965-1748(02)00100-5 12429113

[B13] Gao Q. Bidochka M. J. Thompson G. J. (2011). Effect of Group Size and Caste Ratio on Individual Survivorship and Social Immunity in a Subterranean Termite. acta Ethol. 15 (1), 55–63. 10.1007/s10211-011-0108-7

[B14] Gao Q. Tancredi S. E. Thompson G. J. (2012). Identification of Mycosis-Related Genes in the Eastern Subterranean Termite by Suppression Subtractive Hybridization. Arch. Insect Biochem. Physiol. 80, 63–76. 10.1002/arch.21026 22549993

[B15] Gao Q. Thompson G. J. (2015). Social Context Affects Immune Gene Expression in a Subterranean Termite. Insect. Soc. 62 (2), 167–170. 10.1007/s00040-015-0389-3

[B16] Hamilton C. Bulmer M. S. (2012). Molecular Antifungal Defenses in Subterranean Termites: RNA Interference Reveals *In Vivo* Roles of Termicins and GNBPs against a Naturally Encountered Pathogen. Dev. Comp. Immunol. 36 (2), 372–377. 10.1016/j.dci.2011.07.008 21824492

[B17] He S. Johnston P. R. Kuropka B. Lokatis S. Weise C. Plarre R. (2018). Termite Soldiers Contribute to Social Immunity by Synthesizing Potent Oral Secretions. Insect Mol. Biol. 27 (5), 564–576. 10.1111/imb.12499 29663551

[B18] Hussain A. Li Y.-F. Cheng Y. Liu Y. Chen C.-C. Wen S.-Y. (2013). Immune-related Transcriptome of *Coptotermes Formosanus* Shiraki Workers: the Defense Mechanism. PLoS One 8 (7), e69543. 10.1371/journal.pone.0069543 23874972 PMC3712931

[B19] Husseneder C. (2010). Symbiosis in Subterranean Termites: a Review of Insights from Molecular Studies. Environ. Entomol. 39 (2), 378–388. 10.1603/EN09006 20388266

[B20] Kaji T. Keiler J. Bourguignon T. Miura T. (2016). Functional Transformation Series and the Evolutionary Origin of Novel Forms: Evidence from a Remarkable Termite Defensive Organ. Evol. Dev. 18 (2), 78–88. 10.1111/ede.12179 26766508

[B21] Lamberty M. Zachary D. Lanot R. Bordereau C. Robert A. Hoffmann J. A. (2001). Insect Immunity. J. Biol. Chem. 276 (6), 4085–4092. 10.1074/jbc.M002998200 11053427

[B22] Lee K. H. Shin S. Y. Hong J. E. Yang S.-T. Kim J. I. Hahm K.-S. (2003). Solution Structure of Termite-Derived Antimicrobial Peptide, Spinigerin, as Determined in SDS Micelle by NMR Spectroscopy. Biochem. Biophysical Res. Commun. 309 (3), 591–597. 10.1016/j.bbrc.2003.08.043 12963031

[B23] Liu L. Li G. Sun P. Lei C. Huang Q. (2015). Experimental Verification and Molecular Basis of Active Immunization against Fungal Pathogens in Termites. Sci. Rep. 5, 15106. 10.1038/srep15106 26458743 PMC4602225

[B24] Liu L. Wang W. Liu Y. Sun P. Lei C. Huang Q. (2019a). The Influence of Allogrooming Behavior on Individual Innate Immunity in the Subterranean TermiteReticulitermes chinensis(Isoptera: Rhinotermitidae). J. Insect Sci. 19 (1), 6. 10.1093/jisesa/iey119 PMC6334631 30649425

[B25] Liu L. Zhao X.-Y. Tang Q.-B. Lei C.-L. Huang Q.-Y. (2019b). The Mechanisms of Social Immunity against Fungal Infections in Eusocial Insects. Toxins 11 (5), 244. 10.3390/toxins11050244 PMC6563085 31035652

[B26] Livak K. J. Schmittgen T. D. (2001). Analysis of Relative Gene Expression Data Using Real-Time Quantitative PCR and the 2−ΔΔCT Method. Methods 25 (4), 402–408. 10.1006/meth.2001.1262 11846609

[B27] Matsuura K. Tamura T. Kobayashi N. Yashiro T. Tatsumi S. (2007). The Antibacterial Protein Lysozyme Identified as the Termite Egg Recognition Pheromone. PLoS One 2 (8), e813. 10.1371/journal.pone.0000813 17726543 PMC1950569

[B28] Mitaka Y. Kobayashi K. Matsuura K. (2017a). Caste-, Sex-, and Age-dependent Expression of Immune-Related Genes in a Japanese Subterranean Termite, *Reticulitermes Speratus* . PLoS One 12 (4), e0175417. 10.1371/journal.pone.0175417 28410430 PMC5391962

[B29] Mitaka Y. Mori N. Matsuura K. (2017b). Multi-functional Roles of a Soldier-specific Volatile as a Worker Arrestant, Primer Pheromone and an Antimicrobial Agent in a Termite. Proc. R. Soc. B 284, 20171134. 10.1098/rspb.2017.1134 PMC5543234 28747483

[B30] Qi G. Bidochka M. J. Thompson G. J. (2012). Effect of Group Size and Caste Ratio on Individual Survivorship and Social Immunity in a Subterranean Termite. Acta Ethologica 15 (1), 55–63. 10.1007/s10211-011-0108-7

[B31] Rath A. C. Andrew C. (2000). The Use of Entomopathogenic Fungi for Control of Termites. Biocontrol Sci. Technol. 10 (5), 563–581. 10.1080/095831500750016370

[B32] Robson S. K. A. Traniello J. F. A. (2016). Division of Labor in Complex Societies: a New Age of Conceptual Expansion and Integrative Analysis. Behav. Ecol. Sociobiol. 70 (7), 995–998. 10.1007/s00265-016-2147-6

[B33] Rosengaus R. B. Reichheld J. L. (2016). Phenoloxidase Activity in the Infraorder Isoptera: Unraveling Life-History Correlates of Immune Investment. Naturwissenschaften 103 (1-2), 14–10. 10.1007/s00114-016-1338-3 26838762

[B34] Rosengaus R. B. Maxmen A. B. Coates L. E. Traniello J. F. A. (1998). Disease Resistance: a Benefit of Sociality in the Dampwood Termite *Zootermopsis Angusticollis* (Isoptera: Termopsidae). Behav. Ecol. Sociobiol. 44 (2), 125–134. 10.1007/s002650050523

[B35] Rosengaus R. B. Lefebvre M. L. Traniello J. F. A. (2000). Inhibition of Fungal Spore Germination by Nasutitermes: Evidence for a Possible Antiseptic Role of Soldier Defensive Secretions. J. Chem. Ecol. 26 (1), 21–39. 10.1023/A:1005481209579

[B36] Rosengaus R. B. Cornelisse T. Guschanski K. Traniello J. F. A. (2006). Inducible Immune Proteins in the Dampwood Termite *Zootermopsis Angusticollis* . Naturwissenschaften 94 (1), 25–33. 10.1007/s00114-006-0151-9 16953417

[B37] Rosengaus R. B. Traniello J. F. A. Bulmer M. S. (2010). “Ecology, Behavior and Evolution of Disease Resistance in Termites,” in Biology of Termites: A Modern Synthesis. Editors Bignell D. Roisin Y. Lo N. (Dordrecht: Springer), 165–191. 10.1007/978-90-481-3977-4_7

[B38] Rosengaus R. Traniello J. (2001). Disease Susceptibility and the Adaptive Nature of Colony Demography in the Dampwood Termite *Zootermopsis Angusticollis* . Behav. Ecol. Sociobiol. 50 (6), 546–556. 10.1007/s002650100394

[B39] Šobotník J. Jirošová A. Hanus R. (2010). Chemical Warfare in Termites. J. Insect Physiology 56 (9), 1012–1021. 10.1016/j.jinsphys.2010.02.012 20223240

[B40] Stroeymeyt N. Grasse A. V. Crespi A. Mersch D. P. Cremer S. Keller L. (2018). Social Network Plasticity Decreases Disease Transmission in a Eusocial Insect. Science 362 (6417), 941–945. 10.1126/science.aat4793 30467168

[B41] Thompson G. J. Crozier Y. C. Crozier R. H. (2003). Isolation and Characterization of a Termite Transferrin Gene Up-Regulated on Infection. Insect Mol. Biol. 12 (1), 1–7. 10.1046/j.1365-2583.2003.00381.x 12542630

[B42] Tian L. Zhou X. (2014). The Soldiers in Societies: Defense, Regulation, and Evolution. Int. J. Biol. Sci. 10 (3), 296–308. 10.7150/ijbs.6847 24644427 PMC3957085

[B43] Yanagawa A. Shimizu S. (2007). Resistance of the Termite, *Coptotermes Formosanus* Shiraki to Metarhizium Anisopliae Due to Grooming. BioControl 52 (1), 75–85. 10.1007/s10526-006-9020-x

[B44] Yanagawa A. Yokohari F. Shimizu S. (2008). Defense Mechanism of the Termite, *Coptotermes Formosanus* Shiraki, to Entomopathogenic Fungi. J. Invertebr. Pathology 97 (2), 165–170. 10.1016/j.jip.2007.09.005 17949740

[B45] Yanagawa A. Yokohari F. Shimizu S. (2009). The Role of Antennae in Removing Entomopathogenic Fungi from Cuticle of the Termite,Coptotermes Formosanus. J. Insect Sci. 9, 1–9. 10.1673/031.009.0601 PMC3011873 19611249

[B46] Yanagawa A. Yokohari F. Shimizu S. (2010). Influence of Fungal Odor on Grooming Behavior of the Termite,Coptotermes Formosanus. J. Insect Sci. 10, 1–14. 10.1673/031.010.14101 21073347 PMC3016997

[B47] Yanagawa A. Fujiwara-Tsujii N. Akino T. Yoshimura T. Yanagawa T. Shimizu S. (2011). Behavioral Changes in the Termite, *Coptotermes Formosanus* (Isoptera), Inoculated with Six Fungal Isolates. J. Invertebr. Pathology 107 (2), 100–106. 10.1016/j.jip.2011.03.003 21414322

[B48] Zeng Y. Hu X. P. Suh S.-J. (2016). Characterization of Antibacterial Activities of Eastern Subterranean Termite, *Reticulitermes Flavipes*, against Human Pathogens. PLoS One 11 (9), e0162249. 10.1371/journal.pone.0162249 27611223 PMC5017719

