# Supplementary material for: A High Soldier Proportion Encouraged the Greater Antifungal Immunity in a Subterranean Termite
Source: Front Physiol. 2022 Jun 6;13:906235. doi: 10.3389/fphys.2022.906235 (PMC9207448; doi:10.3389/fphys.2022.906235)
Supplement: Supplementary file 1 [file DataSheet1.docx]

**Supplementary information**

**A high soldier-proportion encouraged the greater antifungal immunity in a subterranean termite**

Wenhui Zeng ^1^, Danni Shen ^1^, Yong chen ^1^, Shijun Zhang ^1^, Wenjing Wu ^1^, Zhiqiang Li ^1,^*

Affiliation 1: Guangdong Key Laboratory of Animal Conservation and Resource Utilization, Guangdong Public Laboratory of Wild Animal Conservation and Utilization, Institute of Zoology, Guangdong Academy of Sciences. No. 105, Xingang Xi Road, Guangzhou, 510260, P. R. China

*Corresponding author: Zhiqiang Li; *e*-mail address: [lizq@giz.gd.cn](mailto:lizq@giz.gd.cn);

**Fig. S1** Survivorship of the Tween-80 treated (control) worker (A) and solider (B) of *Coptotermes formosanus* in different compositions of the worker and soldier ratio under fixed group size (n = 20). The numbers in the legend denote the specific number of workers and soldiers. The P-values were calculated using the Log-rank (Mantel—Cox) test (*p* < 0.05).
